# The low expression of miR-451 predicts a worse prognosis in non-small cell lung cancer cases

**DOI:** 10.1371/journal.pone.0181270

**Published:** 2017-07-12

**Authors:** Akiteru Goto, Masamitsu Tanaka, Makoto Yoshida, Michinobu Umakoshi, Hiroshi Nanjo, Kouya Shiraishi, Motonobu Saito, Takashi Kohno, Sei Kuriyama, Hayato Konno, Kazuhiro Imai, Hajime Saito, Yoshihiro Minamiya, Daichi Maeda

**Affiliations:** 1 Department of Cellular and Organ Pathology, Graduate School of Medicine, Akita University, Akita, Japan; 2 Department of Molecular Medicine and Biochemistry, Graduate School of Medicine, Akita University, Akita, Japan; 3 Department of Clinical Pathology, Akita University Hospital, Akita, Japan; 4 Division of Genome Biology, National Cancer Center Research Institute, Tokyo, Japan; 5 Department of Thoracic Surgery, Akita University Hospital, Akita, Japan; University of South Alabama Mitchell Cancer Institute, UNITED STATES

## Abstract

**Purpose:**

miR-451 is a tumor suppressive microRNA with several target genes, including *Macrophage migration inhibitory factor* (*MIF*). As little is known about the expression and clinicopathological significance of mir-451 in NSCLC, we performed a clinicopathological study of 370 NSCLC cases to clarify them. Cell biological experiments were also performed on NSCLC cell lines to confirm the tumor-suppressive role of miR-451 and whether or not *MIF* is targeted by miR-451.

**Methods:**

We analyzed 370 NSCLC cases for the miR-451 expression by quantitative real-time polymerase chain reaction and the MIF expression by immunohistochemistry. Eighty-four background lung tissue samples were also evaluated for the miR-451 expression. The clinicopathological and genetic factors surveyed were the disease-free survival, smoking status, histological type, disease stage, *EGFR* gene mutations and *ALK* rearrangements. In 286 adenocarcinoma cases, the invasive status (adenocarcinoma *in situ*, minimally invasive adenocarcinoma and invasive adenocarcinoma) was also evaluated. Five NSCLC cell lines (H23, H441, H522, H1703, and H1975) were cultured and evaluated for their miR-451 and MIF expression. The cell lines with lower miR-451 and higher MIF expressions were then selected and transfected with miR-451-mimic to observe its effects on MIF expression, Akt and Erk status, cell proliferation, and cell migration.

**Results:**

The miR-451 expression was down-regulated in cancer tissues compared with background lung tissues (*P*<0.0001). Factors such as advanced disease stage, positive pleural invasion and nodal status and being a smoker were significantly correlated with a lower expression of miR-451 (*P*<0.05 each), while *EGFR* gene mutations and *ALK* rearrangements were not. In adenocarcinoma, invasive and minimally invasive adenocarcinoma showed lower expression of miR-451 than adenocarcinoma *in situ* (*P*<0.0005, respectively). A survival analysis showed that a lower expression of miR-451 was an independent predictor of a poor prognosis for NSCLC (*P*<0.05). The MIF expression was inversely correlated with the miR-451 expression. Out of 5 NSCLC cell lines examined, H441 and H1975 showed higher MIF and lower miR-451 expressions. After the transfection of miR-451-mimic, the MIF expression and phosphorylated Akt expression of these cell lines was suppressed, as were cell proliferation and cell migration.

**Conclusion:**

This clinicopathological study of 370 NSCLC cases and the cell biological studies of NSCLC cell lines clarified the tumor-suppressive role of miR-451 and its prognostic value. We also validated *MIF* as a target of miR-451 in NSCLC.

## Introduction

By specifically binding to the complimentary sequence in the 3’ untranslated region (3’-UTR) of messenger RNAs (mRNAs), microRNAs (miRNA), small non-coding single-strand RNAs 17 to 28 nucleotides in length, suppress the translation or accelerate the degradation of their target mRNAs [1,2,3]. Besides their roles in a variety of fundamental biological processes, such as cell development, differentiation, proliferation and apoptosis, miRNAs have also been shown to be important factors in the development of various cancer types, including lung, breast and colorectal cancers [1,3–7].

Among the miRNAs involved in carcinogenesis and the development of various cancers, miR-451, located on chromosome 17 at 17q11.2, is of note because of its suppressive role upon several malignant features of cancer, including tumor growth, invasion, radioresistance, and chemoresistance [8–15]. As a result, a lower expression of miR-451 is correlated to a worse prognosis in gastric cancer, hepatocellular cancer, esophageal squamous cancer and nasopharyngeal carcinoma [12, 13]. In non-small cell lung cancer (NSCLC), multiple studies using NSCLC cell lines have indicated the tumor-suppressive role of miR-451. The upregulation of miR-451 inhibits growth and enhances apoptosis of the NSCLC cell line A549, sensitizing it to cisplatin and irradiation [8, 14]. Furthermore, the re-expression of miR-451 can reverse the epithelial-mesenchymal transition (EMT) to mesenchymal-epithelial transition (MET) and inhibit the invasion and metastasis of docetaxel-resistant lung adenocarcinoma (LAD) cells [10]. However, despite mounting evidence suggesting the tumor-suppressive role of miR-451 in NSCLC, only a few studies have addressed its prognostic and clinicopathological roles in a clinical setting [15].

We therefore examined the miR-451 expression in NSCLC patients and conducted detailed analyses to clarify its clinicopathological and prognostic role. In analyses of the lung adenocarcinoma group, we attempted to strengthen the quality of the study by incorporating newly advocated histopathological prognostic factors, such as spread through air spaces (STAS) and nuclear and mitotic grade risk stratification, into the comparative factors [16,17]. In addition, we performed cell biological experiments on NSCLC cell lines to confirm the effect of miR-451 on cell proliferation and migration.

miRNAs exert their biological roles of silencing or repressing their target genes by forming an RNA-induced silencing complex (RISC) with specific mRNAs having complimentary sequences in their 3’-UTR [2]. For mir-451, several genes have been validated as its targets in cancer, including *c-myc*, *ras-related protein 14 (RAB14)*, *MDR-1* and *Macrophage migration inhibitory factor* (*MIF*) [11, 15, 18, 19]. Among them, MIF is notable, as it was validated as a target gene of mir-451 in different types of cancers, including gastric cancer, colon cancer, nasopharyngeal carcinoma and osteosarcoma [12, 18, 19]. MIF is a glycoprotein originally isolated from the supernatants of activated T lymphocyte culture, and its role as a pro-inflammatory cytokine or chemokine has been reported [20]. MIF also accelerates tumor growth by activating the MAPK/PI3K/Akt pathways, promoting tumor-associated angiogenesis and inhibiting the antitumor immune response through the autocrine or paracrine mechanism [20]. Indeed, its expression was found to be increased in NSCLC tissues, and a high MIF expression was associated with a poor prognosis for NSCLC [21].

Given the evidence suggesting that MIF is targeted by miR-451 and involved in NSCLC development, we performed MIF immunohistochemistry and cell biological experiments to determine whether or not MIF is indeed targeted by miR-451 in NSCLC.

## Materials and methods

### NSCLC cases and classification

We analyzed 370 NSCLC cases. The clinicopathological characteristics such as the age, sex, Brinkman index and survival, were obtained from clinical records. These 370 NSCLC cases were retrieved from surgical specimens obtained between 2005 and 2014 at Akita University Hospital (Akita, Japan). None of the patients had received preoperative chemotherapy. Participants were between 30 and 86 years of age. The histopathological diagnoses were made according to the World Health Organization Classifications [22] and graded according to the general rule for clinical and pathological records of lung cancer by The Japanese Lung Cancer Society (G1, well-differentiated; G2, moderately differentiated; G3, poorly differentiated; G4, large cell carcinoma) [23]. The presence of pleural invasion was assessed with Elastica-Masson staining of the samples. Adenocarcinomas were classified into adenocarcinoma *in situ* (AIS), minimally invasive adenocarcinoma (MIA) and invasive adenocarcinoma (IA) [22]. IAs were further classified into subtypes according to their predominant histological patterns [22]. For adenocarcinomas, the spread through air spaces (STAS) was also evaluated, and nuclear and mitotic grade risk stratification was performed, as these factors are reportedly associated with the prognosis [16], [17]. The disease stage of the cases was determined according to the UICC TNM classification [24]. The disease-free survival was measured from the date of surgical resection to the date of recurrence or death due to NSCLC or the date when the patients were last known to be alive. Ethical approval was obtained from Akita University, Faculty of Medicine, Ethics Committee (Reference No.1241), as was written informed consent from each patient.

### An miR-451 expression analysis by quantitative real-time polymerase chain reaction (qRT-PCR)

RNA was extracted from FFPE tumor tissues 10 μm in thickness taken from each case using the RecoverAll Total Nucleic Acid Isolation kit (Life Technologies, Carlsbad, CA, USA). RNA extraction was also performed on FFPE background lung tissues from 84 adenocarcinoma cases resected in 2005 and 2014. The expression of mature miR-451 was determined by qRT-PCR as described in detail previously using the TaqMan Human MicroRNA Assay kit (Assay ID #001141; Life Technologies) and the 7900 HT-Fast real-time PCR system (Applied Biosystems, Carlsbad, CA, USA) [3]. RNU6B was used as an endogenous control (#4440887; Life Technologies). All assays were performed in triplicate. The miRNA expression was quantified as δCt values, where Ct = threshold cycle, δCt = (Ct target microRNA − Ct RNU6B). δCt was calculated using the RQ manager software program, version 1.2 (Applied Biosystems).

### Analyses of EGFR gene mutations and ALK rearrangements

For *EGFR* mutation analysis, DNA was extracted from 10-μm-thick FFPE or frozen tumor tissue from each case using the Allprep DNA/RNA Micro kit (QIAGEN, Hilden, Germany) DNA samples were screened for somatic mutations in *EGFR* exons 19 and 21 by a high-resolution melting (HRM) analysis, as described elsewhere [25]. The HRM analysis was carried out using primer set A for the detection of *EGFR* mutations in exon 19 and primer set B for the detection of EGFR mutations in exon 21. The sequences of primer set A were 5’-AAAATTCCCGTCGCTATC-3’ (forward) and 5’-AAGCAGAAACTCACATCG-3’ (reverse). The sequences of primer set B were 5’-AGATCACAGATTTTGGGC-3’ (forward) and 5’-ATTCTTTCTCTTCCGCAC-3’ (reverse). For the *ALK* rearrangement analysis, total RNA was extracted from frozen tumor tissue from each case using the Allprep DNA/RNA Micro kit (QIAGEN). Reverse transcription-PCR was performed using SuperScript III Reverse Transcriptase (Thermo Fisher Scientific, Waltham, MA, USA) [26].

### MIF immunohistochemistry (IHC)

A total of 110 cases were arbitrarily chosen for MIF IHC (87 adenocarcinoma, 20 squamous cell carcinoma, 3 large-cell carcinoma). Sections 4-μm-thick were stained by a Ventana Discovery XT® autostainer (Ventana Medical Systems, Tucson, AZ, USA) using a mouse antibody against MIF (clone # 932612; R&D Systems, Minneapolis, MN, USA) at 8 μg/ml. The numbers of stained cells were scored using a proportion score (PS): 0 (0% cells), 1+ (<10% cells positive), 2+ (10%-50% cells positive), or 3+ (>50% cells positive). The intensity score (IS) was also scored according to a numerical scale (0: no expression; 1: weak; 2: moderate, 3: strong expression). The PS (0–3) and IS (0–3) were summed as the total score (TS) from 0 to 6 [27]. Human ovarian tissues were utilized as positive controls. Negative controls were obtained by omitting the primary antibody.

### Cell lines and cell culture

Five NSCLC cell lines (H23, H441, H522, H1703, and H1975) were obtained from the American Type Culture Collection (ATCC) by Dr. Takashi Kohno (National Cancer Center Research Institute Tokyo, Japan) and gifted. These cell lines were cultured in RPMI1640 with 10% FBS (Thermo Fisher Scientific, Waltham, MA, USA) and 1% penicillin-streptomycin (P/S; Nacalai Tesque, Kyoto, Japan) in a humidified atmosphere with 5% CO_2_ and 95% air. Small RNA was isolated using miRNeasy mini kit (Qiagen, Venio, Netherlands).

### Western blot analyses

Cells were lysed in buffer containing 50 mM Tris-HCl, pH 7.6, 150 mM NaCl, 0.1% SDS, 1% Nonidet P-40, and 0.5% sodium-deoxycholate. Ten μg of proteins were separated by gel electrophoresis on 10% gels, transferred to nitrocellulose membranes, and detected by immunoblotting using a chemiluminescence system (GE Healthcare Japan, Tokyo, Japan). Antibodies used for western blot as follows: MIF (clone # 932612; R&D Systems, 1μg/ml),　α-tubulin (clone; B-5-1-2, Sigma-Aldrich, St. Louis, MO, USA, 0.2μg/ml). Antibodies against Phosphorylated Akt (Thr308), Akt, Phosphorylated Erk1/2 (Thr202/Tyr204) and Erk1/2 were purchased from Cell Signaling (Danvers, MA, USA, 0.1μg/ml).

### Oligonucleotide transfection, cell proliferation, and cell migration assays

H-441 and H-1975 were transfected with the oligonucleotide (miRIDIAN microRNA Human hsa-miR-451a-Mimic or miRIDIAN microRNA Mimic Negative Control #1, GE Healthcare Japan) at a final concentration of 200 nM using LipofectAMINE RNAiMAX reagent (Thermo Fisher Scientific). To evaluate the cell proliferation, H-441 and H-1975 cells with transfection of miR-451 mimic or miR mimic negative control were seeded into 6-well plates (2.5 x 10^4^/well). Cells were trypsinized at various time points and non-apoptotic cells were counted. The data points indicate the average results from three dishes. Migration assays were performed using transwell chambers with a polycarbonate nucleopore membrane (8 μm pore size, BD Falcon). Test cells (2 × 10^4^) in medium containing 0.2% FBS were seeded into the upper chambers of the transwells. The lower compartments were filled with the same medium containing 10% FBS. Cells that had migrated to the undersurface of the membrane after 24h of incubation were fixed with 4% paraformaldehyde and stained with Giemsa solution. The number of migrating cells was determined by counting cells in five microscopic fields per well, and the extent of migration was expressed as the average number of cells per field. The assays were performed three times.

### Statistical analyses

Statistical analysis was performed with the GraphPad Prism 6 software program for Windows (GraphPad Software, San Diego, CA, USA) except for the multivariate survival analysis, which was performed with the JMP 13.1 software program (SAS Institute Japan, Tokyo, Japan). Differences in the miRNA expression among the groups were analyzed by paired or unpaired t-tests and a one-way analysis of variance (one-way ANOVA). The chi-squared test was used to evaluate the clinicopathological or genetic correlations. Survival curves were estimated by the Kaplan–Meier method, and the differences in the disease-related survival between subgroups were compared by the log-rank test. The Cox proportional hazards model was used for the multivariate survival analysis. Differences were determined to be significant when the probability value (*p*) was <0.05.

## Results

### Histopathological classification, clinicopathological factors and genetic alterations of the cases

The cases were 286 adenocarcinomas, 73 squamous cell carcinomas, 5 adenosquamous carcinomas, 4 large cell carcinomas and 2 mucoepidermoid carcinomas. Regarding grading, 132 cases were classified as grade 1, 115 as grade 2, 119 as grade 3 and 4 as grade 4. Pleural invasion was noted in 145 cases. Of the 286 adenocarcinoma cases, 28 were AIS, 25 were MIA and 233 were IA. All of the AIS and MIA cases were non-mucinous adenocarcinomas. The IA cases comprised 11 lepidic, 45 acinar, 128 papillary, 36 solid and 13 mucinous adenocarcinomas. Of the 286 adenocarcinoma cases, 44 showed STAS, and 78 were categorized as high-risk cases by nuclear and mitotic grade risk stratification. According to the TNM classification, 148 cases were stage 0 or Ia disease, 103 were stage Ib, 62 were stage II and 59 were stage III or IV, with 89 cases having metastases to the lymph nodes.

Of the 370 total cases, 164 were never-smokers, and 206 were former and current smokers. The Brinkman index for former and current smokers ranged from 115–3600 with an average of 1063.7.

An *EGFR* mutation analysis was successfully performed in 296 cases (230 adenocarcinomas, 59 squamous cell carcinomas, 4 adenosquamous carcinomas, 3 large-cell carcinomas). Eighty (27.0%) harbored *EGFR* mutations. Histopathologically, 78 of the 80 *EGFR*-mutated cases were adenocarcinoma, and the rest were adenosqumaous carcinoma (n = 1) and squamous cell carcinoma (n = 1). An *ALK* rearrangement analysis was performed in 195 cases (140 adenocarcinomas, 50 squamous cell carcinomas, 3 adenosquamous carcinomas, 2 large-cell carcinomas). Six (3.1%) were positive for *ALK* rearrangement. All of the cases with *ALK* rearrangement were adenocarcinomas. An EGFR mutation analysis was not successfully performed in 74 cases due to the poor quality of DNA extracted from FFPE samples and/or failures of the HRM analysis. ALK analyses were not performed in 175 cases because frozen samples were not available.

### Differences in the miR-451 expression between background lung tissue and NSCLC

**Fig 1:**
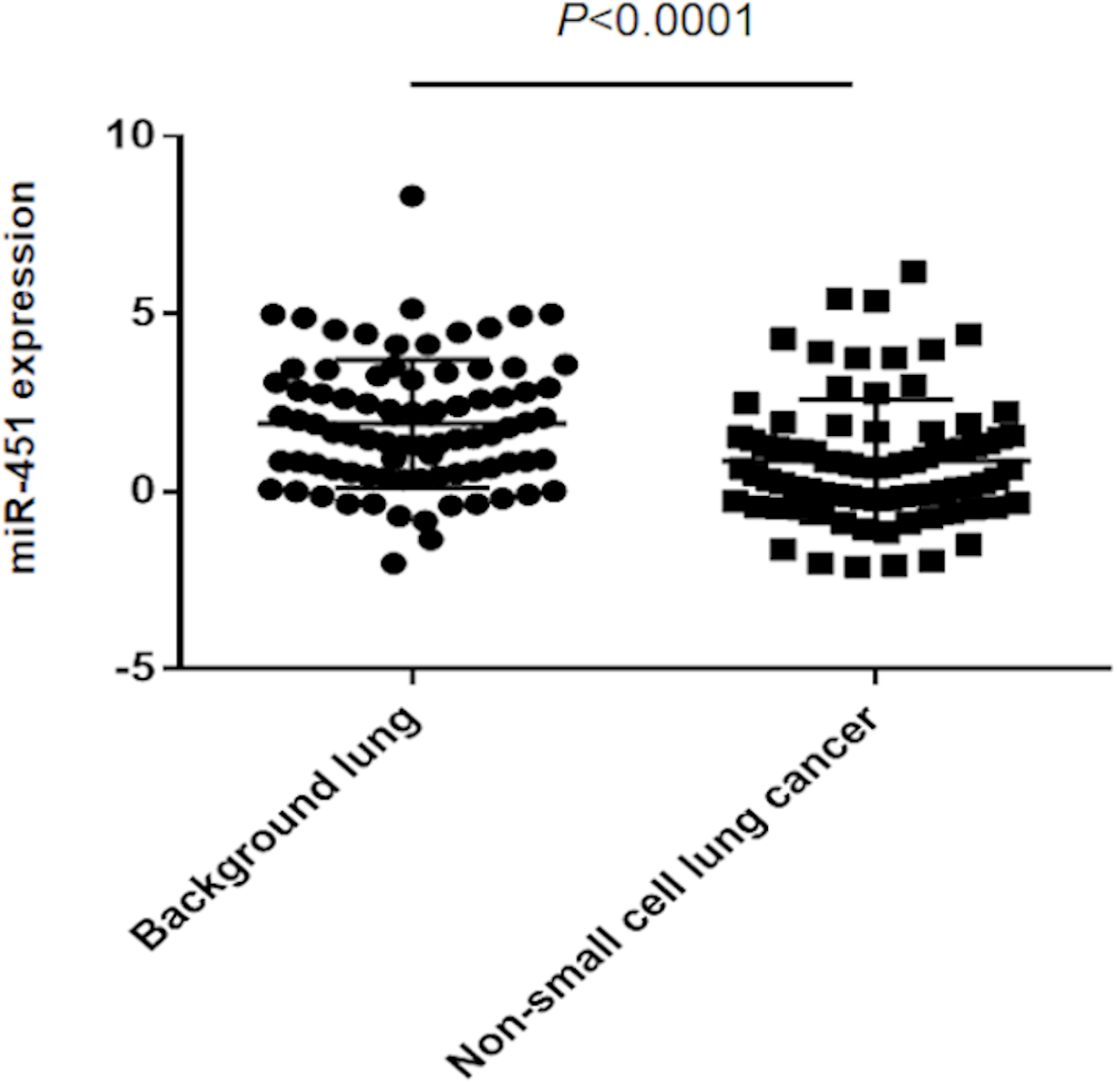
miR-451 expression in paired background lung and non-small cell lung cancer tissues. The miR-451 expression is significantly lower in NSCLC tissues than in background lung tissue (*P*<0.0001 with paired t-test). Y-axis: -δCt = - -(Ct miR-451−Ct RNU6B).

Eighty-four pairs of background lung tissue and NSCLC (72 adenocarcinomas and 12 squamous cell carcinomas) were compared for their miR-451 expression. The average -δCt value (-Ct miR-451+Ct RNU6B) for background lung tissue was 1.93 versus 0.86 for NSCLC (Fig 1). Thus, the miR-451 expression in NSCLC was significantly downregulated in comparison with the background lung tissue by a paired *t*-test (*P*<0.0001).

### miR-451 expression and clinicopathological factors in NSCLC

In NSCLC, factors such as male gender; stages IB, II, III and IV disease; histological grade of G2, G3 and G4; positive pleural invasion and nodal status and being a smoker were significantly correlated with a lower expression of miR-451 (*P*<0.05, respectively) (Table 1). Squamous cell carcinoma showed a lower expression of mir-451 than adenocarcinoma (*P*<0.05). In contrast, the *EGFR* gene or *ALK* rearrangement status was not correlated with the miR-451 expression.

**Table 1:** Correlations of the miR-451 expression with clinicopathological factors in non-small cell lung carcinomas.

		Number of cases	miR-451 expression*	*P*-value
Gender	Male	236	0.606	
	Female	134	1.037	<0.05
Age, years	>65	114	0.907	
	≤65	256	0.699	0.307
Histological type	Adenocarcinoma	286	0.906**,***	
	Squamous cell carcinoma	73	0.285**,***	<0.05***
	Adenosquamous carcinoma	5	0.652**	
	Large cell carcinoma	4	0.704**	
	Mucoepidermoid carcinoma	2	0.568**	
Stage	Ia	148	1.31	
	Ib, II, III, IV	222	0.395	<0.000001
Histological grade	G1	132	1.22	
	G2,G3, G4	238	0.51	<0.001
Pleural invasion	negative	226	0.928	
	positive	144	0.5	<0.05
Lymph node metastasis	negative	281	0.866	
	positive	89	0.425	<0.05
Smoking status	Never-smoker	164	1.034	
	Smoker	206	0.5784	<0.00001
*EGFR*gene status	wild type	216	0.631	
	mutant	80	0.729	0.675
*ALK r*earrangement	negative	188	0.733	
	positive	6	1.05	0.938

*Average -δCt = - (Ct miR-451−Ct RNU6B)

**P<0.0001 with a one-way analysis of variance (ANOVA) test

***P<0.05 with an unpaired t-test

In lung adenocarcinoma, the miR-451 expression was lower in MIA and IA than in AIS (*P*<0.0005, respectively) (Table 2). In IA, no significant difference was observed among the histological types. The miR-451 expression was also lower in high-grade cases than in low-grade cases by nuclear and mitotic grade risk stratification (*P*<0.05), while the STAS status was not correlated with the miR-451 expression.

**Table 2:** Correlations of the miR-451 expression with clinicopathological factors in lung adenocarcinomas.

		Number of cases	miR-451 expression*	*P*-value
Adenocarcinoma progression	Adenocarcinoma *in situ* (AIS)	28	2.54**,***	<0.0001**
	Minimally invasive adenocarcinoma (MIA)	25	1.02**,***	<0.0005***
	Invasive adenocarcinoma (IA)	233	0.697**,***	
Histological type of invasive adenocarcinoma	Lepidic adenocarcinoma	11	0.452	0.1363
	Acinar adenocarcinoma	45	1.03	
	Papillary adenocarcinoma	128	0.81	
	Solid adenocarcinoma	36	0.123	
	Mucinous adenocarcinoma	13	0.247	
Spread through air spaces (STAS)	Absent	242	0.935	
	Present	44	0.708	0.443
Nuclear and mitotic grade risk stratification	Low grade	208	1.051	
	High grade	78	0.504	<0.05

*Average -δCt = - (Ct miR-451−Ct RNU6B)

**P<0.0001 with a one-way analysis of variance (ANOVA) test

***P<0.0005 with an unpaired t-test in comparison with AIS

### Prognostic significance of the miR-451 expression in NSCLC and lung adenocarcinoma

Cases were divided into miR-451 high-expression (H) and low-expression (L) groups based on the average miR-451 expression of the cohort. For NSCLC, the average miR-451 expression (-δCt value [-Ct miR-451+Ct RNU6B]) was 0.761, and 173 cases were categorized as miR-451 H and 197 cases as miR-451 L. For adenocarcinoma, the average miR-451 expression (-δCt value [-Ct miR-451+Ct RNU6B]) was 0.896, and 132 cases were categorized as miR-451 H and 154 cases as miR-451 L.

**Fig 2:**
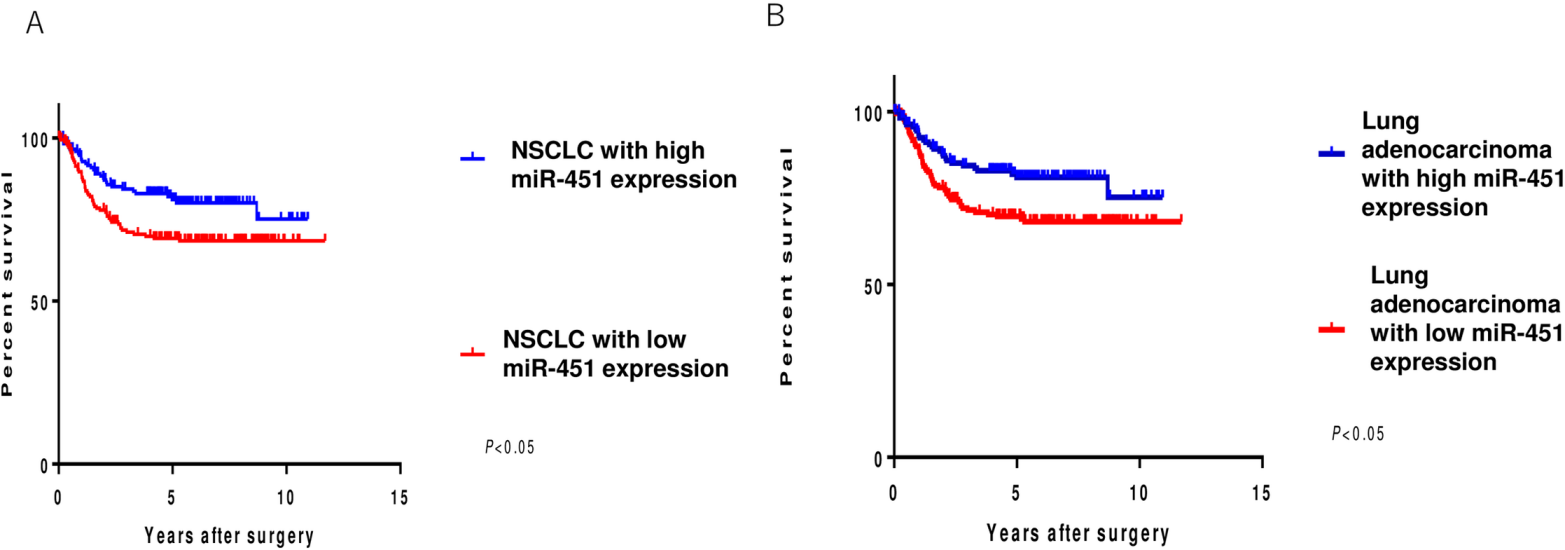
miR-451 and the disease-free survival (DFS) in non-small cell lung cancer (NSCLC) and lung adenocarcinoma cases. (A) NSCLC cases with a low miR-451 expression showed a significantly worse prognosis than cases with a high miR-451 expression. (B) Lung adenocarcinoma cases with a low miR-451 expression showed a significantly worse prognosis than cases with a high miR-451 expression. Tick: censored cases.

In both NSCLC and adenocarcinoma, the miR-451 L group showed a worse disease-free survival than the miR-451 H group (Fig 2). We also analyzed the prognostic value of other clinicopathological factors to identify the factors significantly influencing the survival. The pathological stage, histological grade, pleural invasion and lymph node metastasis were confirmed as factors significantly correlated with the prognosis in NSCLC (Table 3). In adenocarcinoma cases, prognostic significance was confirmed for adenocarcinoma progression, STAS and nuclear and mitotic grade risk stratification (Table 4). We next performed a multivariate survival analysis using these factors as variables (Tables 5 and 6), where the *P-*values for mir-451 expression (H/L) were <0.05 in the NSCLC group and 0.316 in the adenocarcinoma group.

**Table 3:** A univariate analysis of clinicopathological factors for a disease-free survival in non-small cell lung cancer patients.

Clinicopathological factors	*P*-value
Gender (Male vs. Female)	0.6033
Age, years (>64 vs. ≤65)	0.3425
Histological type (Non-adenocarcinoma vs. Adenocarcinoma)	0.0793
Pathological stage (Ib, II, III, and IV vs. 0 and Ia)	**<0.0001**
Histological grade (G2, G3, and G4 vs. G1)	**<0.05**
Pleural invasion (Present vs. Absent)	**<0.0005**
Lymph node metastasis (Positive vs. Negative)	**<0.0001**
Smoking status (Smoker vs. Never-smoker)	0.9318
EGFRgene status (Mutated vs. Wild type)	0.1977
ALK rearrangement (Positive vs. Negative)	0.1905
miR-451 expression (Low vs. High)	**<0.05**

**Table 4:** A univariate analysis of clinicopathological factors for a disease-free survival in non-small cell lung cancer patients.

Clinicopathological factors	*P*-value
Adenocarcinoma progression (Invasive adenocarcinoma vs. Adenocarcinoma *in situ* and minimally invasive adenocarcinoma)	**<0.0001**
Spread through air spaces (STAS) (Present vs. Absent)	**<0.05**
		Nuclear and mitotic grade risk stratification (High-grade vs. Low-grade)	**<0.0001**
miR-451 expression (Low vs. High)	**<0.05**		

**Table 5:** A multivariate survival analysis in non-small cell lung cancer cases.

	HR (95% CI)	*P* value
Pathological stage (Ib, II, III, and IV vs. 0 and Ia)	1.77(0.855–3.71)	0.123
Histological grade (G2, G3, and G4 vs. G1)	1.14(0.639–2.12)	0.659
Pleural invasion (Present vs. Absent)	1.47(0.877–2.54)	0.146
Lymph node metastasis (Positive vs. Negative)	3.21(1.96–5.32)	**<0.0001**
miR-451 expression (Low vs. High)	1.59(1.01–2.56)	**<0.05**

HR, hazard ratio; CI, confidence interval

**Table 6:** A multivariate survival analysis in lung adenocarcinoma cases.

	HR (95% CI)	*P* value
Adenocarcinoma progression (Invasive adenocarcinoma vs. Adenocarcinoma *in situ* and minimally invasive adenocarcinoma)	5.16 (1.54–32.1)	**<0.05**
Spread through air spaces (STAS) (Present vs. Absent)	1.59 (0.895–2.71)	0.095
Nuclear and mitotic grade risk stratification (High-grade vs. Low-grade)	2.59 (1.61–4.20)	**<0.0001**
miR-451 expression (Low vs. High)	1.29 (0.79–2.16)	0.316

HR, hazard ratio; CI, confidence interval

### MIF and miR-451 expression in NSCLC

**Fig 3:**
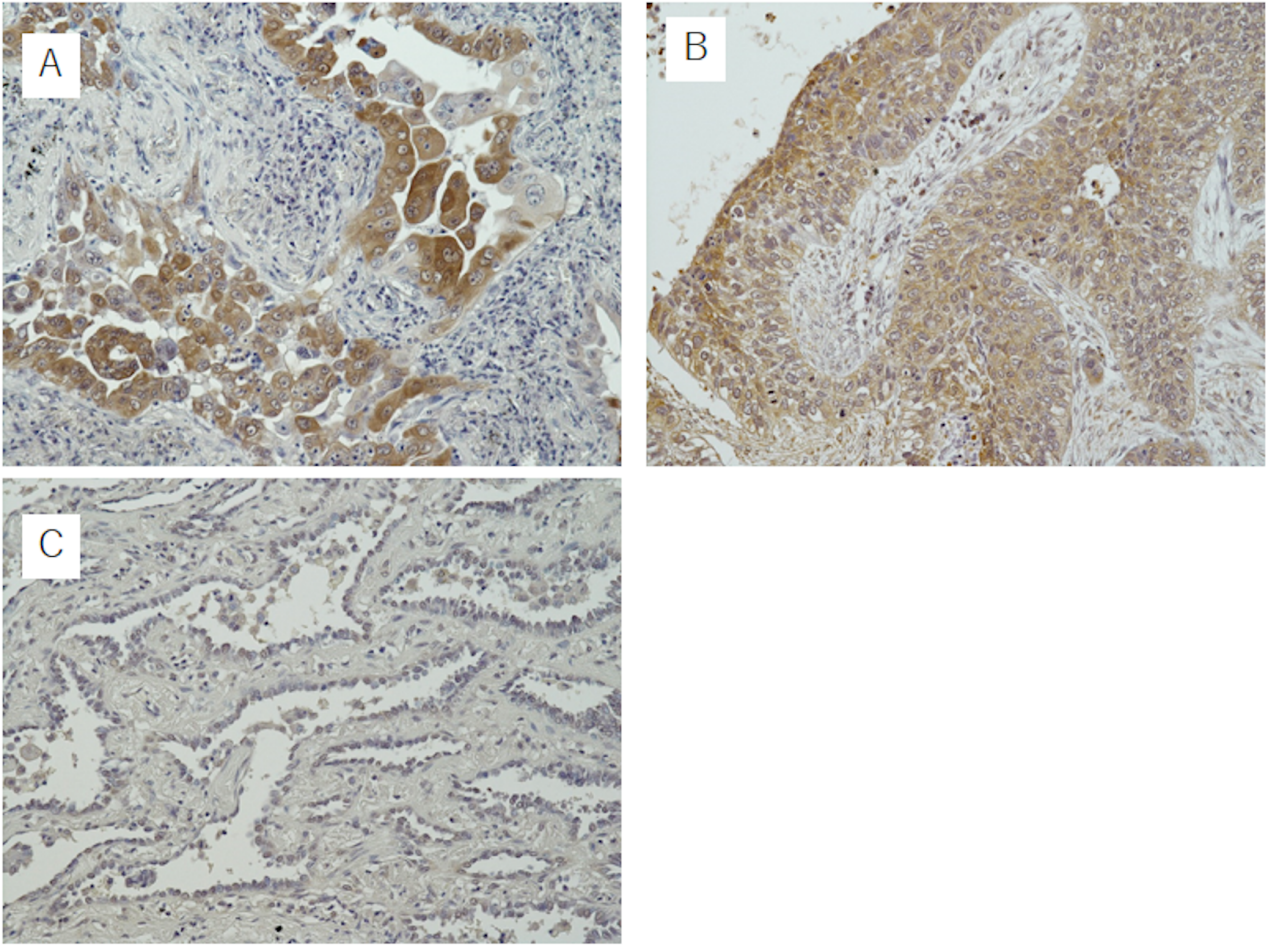
The macrophage migration inhibitory factor (MIF) expression of non-small cell lung cancers. Representative adenocarcinoma (a) and squamous cell carcinoma (b) cases with a high expression of MIF (objective x20). The cases were scored as follows: (a) Proportion score (PS), 3; intensity score (IS), 3 and total score (TS), 6. (b) PS, 3; IS, 2 and TS, 5. An adenocarcinoma case negative for MIF expression (TS, 0) is shown in (c).

Of the 110 NSCLC cases analyzed, 41 were scored as 0 in TS (37.3%), 9 as 2 (8.1%), 13 as 3 (8.1%), 18 as 4 (16.2%), 18 as 5 (16.2%) and 11 as 6 (9.9%) (Fig 3). On categorizing them into an MIF low-expression group (TS 0 and 2), intermediate-expression group (TS 3 and 4) and high-expression group (TS 5 and 6), the mean expression/SEM of miR-451 in each group (-δCt value [-Ct miR-451+Ct RNU6B]) was 2.371/0.3636, 0.2364/0.6034 and -1.744/0.2630, respectively. Thus, the miR-451 expression differed among MIF low-, intermediate-, and high-expression groups (*P*<0.05, one-way ANOVA) and decreased as the MIF expression increased (*P*<0.05, unpaired t-test). Stage 0 and IA cases in the MIF low-expression group (25/50 cases, 50%) were significantly more frequent than in the MIF intermediate- and high-expression groups (12/60 cases, 20%) (*P*<0.01, chi-squared test). There were no significant differences in the prognosis among the MIF expression groups.

### Induction of miR-451 in NSCLC cell lines suppressed MIF expression, cell proliferation, and cell migration

**Fig 4:**
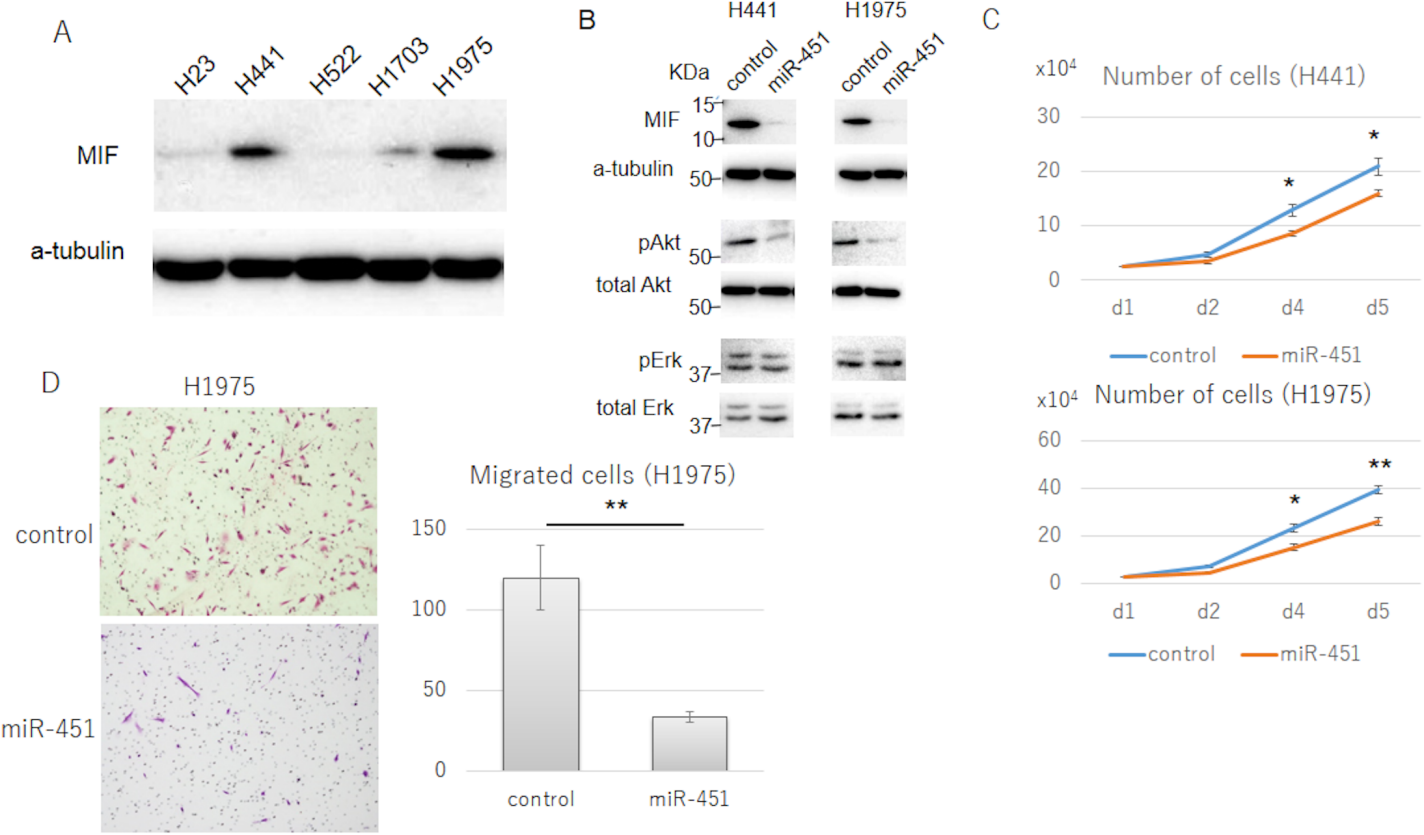
Macrophage migration inhibitory factor (MIF) expression of non-small cell lung cancer (NSCLC) cell lines and the effects of miR-451 mimic transfection of NSCLC cell lines. (A) MIF expression in NSCLC cell lines by western blotting. H441 and H1975 showed relatively higher MIF expression. (B) Western blotting of MIF, phosphorylated Akt (pAkt), total Akt, phosphorylated Erk (pErk), and total Erk of H441 and H1975 cells after miR-451-mimic (miR-451) and Mimic Negative Control (control) transfection. (C) Cell proliferation assay of H441 and H1975 cells after miR-451-mimic (miR-451) and Mimic Negative Control (control) transfection d: day, error bars: +/-SEM, *: *P*<0.05 (unpaired t-test), **: *P* <0.005 (unpaired t-test). (D) Cell migration assay of H1975 cells after miR-451-mimic (miR-451) and Mimic Negative Control (control) transfection. Migrated cell stained with Giemsa solution (left) and migrated cell counts (right). Error bars: +/-SEM, **:*P*<0.005 (unpaired t-test).

Comparing H23, H441, H522, H1703, and H1975 for the expression of MIF showed higher expression in H441 and H1975 and lower for the rest (Fig 4A). The miR-451 expression of the cells was H-23,-3.86; H441,-4.33; H522, -4.08; H1703, -4.94, and H1975,-4.27, respectively (-δCt value [-Ct miR-451+Ct RNU6B]). H441 and H1975 cells, which showed increased expression of MIF and reduced miR-451, were selected for further analyses. H441 and H1975 cells were transfected with an oligonucleotide mimicking miR-451 (miR-451-mimic). After the transfection, the miR-451 expression in these cells was augmented (data not-shown), and the MIF expression was down-regulated (Fig 4B). Induction of miR-451 reduced Akt activation in these cells, which was detected by phosphorylation of Akt at Thr308, while it did not modify Erk activation (Fig 4B). Moreover, miR-451-mimic transfection suppressed the cell proliferation of H441 cells and H1975 cells (Fig 4C), and the cell migration of H1975 cells (Fig 4D).

## Discussion

Accumulating evidence has revealed the diverse roles of miRNA in the development of human cancers [1, 3, 4]. These miRNAs may function as oncogenic factors (e.g. miR-21) or as tumor suppressive factors (e.g. let-7) [3–7]. miR-451 is an miRNA linked to cancer development and is considered a tumor suppressor based on clinicopathological and cell biological evidence. From a clinicopathological perspective, the miR-451 expression is downregulated in various types of cancers, and its lower expression is correlated with a worse prognosis in cancers such as NSCLC, gastric cancer and hepatocellular cancer [12, 13, 15]. From a cell biology perspective, the upregulation of miR-451 inhibits malignant biological behavior, such as chemoresistance, radioresistance, EMT, invasion and metastasis, in multiple cancer cell lines [8–15]. Our clinicopathological observation of 370 cases also clearly illustrated the tumor-suppressive role of miR-451 in NSCLC. miR-451 was downregulated in NSCLC compared to background lung tissue, and a lower expression of miR-451 was an independent predictor of a poor prognosis for NSCLC. Furthermore, its downregulation was associated with multiple clinicopathological factors, such as an advanced disease stage, greater histological grade, pleural invasion and nodal metastasis, all of which were related to a worse biological behavior of tumor. The tumor suppressive role of miR-451 was also illustrated in the lung adenocarcinoma group (286 cases), where the lower expression of mir-451 was also a worse prognostic factor in a univariate survival analysis, and its downregulation was associated with adenocarcinoma progression from AIS and MIA to IA and a high tumor grade on nuclear and mitotic grade risk stratification. By miR-451-mimic transfection, cell biological experiments of multiple NSCLC cell lines also illustrated its tumor-suppressive properties by attenuating cell proliferation and cell migration.

Multiple mechanisms are known to be involved in the regulation of the miR-451 expression, including epigenetic regulation, activation by SMAD3 and SMAD4 and transcriptional activation by transcription factor E2a [13]. The first two factors may be involved in the dysregulation of miR-451 in NSCLC, as they are reportedly perturbed in the carcinogenic process of NSCLC [28, 29]. Of the two, epigenetic regulation is more likely to be involved, based on the present results showing a strong correlation between tobacco smoking and miR-451 downregulation [30]. We previously screened epigenetically silenced microRNAs by the 5-aza-2′-deoxycytidine (5-aza-CdR) treatment of NSCLC cell lines, which failed to show miR-451 as an epigenetically silenced microRNA [31]. However, Wang et al. reported a 2.5-fold increase in the miR-451 expression in NSCLC cell lines by similar treatment with 5-aza-CdR [15]. The discrepancy between these two studies may be attributed to our criteria for selecting epigenetically silenced microRNAs in the microRNA microarray study: (*i*) miRNAs within CpG islands, (*ii*) miRNAs ≤1 kbp downstream of CpG islands and (*iii*) miRNAs within gene introns whose host promoters have CpG islands. miR-451 does not meet the criteria, as it does not have any CpG islands within the region 2 kb upstream. Wang et al. hypothesized the DNA methylation effects of the long-distant (>2 kb) CpG-rich region of miR-451 [15]. Further investigation of NSCLC clinical samples is therefore warranted to clarify this issue by an analysis of the long-distant CpG-rich region of miR-451.

Interestingly, AIS showed higher miR-451 expression than background lung tissue. In this specific early-stage lung adenocarcinoma that retains its miR-451 function, the miR-451 expression is presumably augmented to oppose the oncogenic processes. In bronchoalveolar stem cells (BSCSs), miR-451 was identified as one of the nine most significantly overexpressed microRNAs compared with control lung cells [31]. Although the underlying mechanism is not clear, the overexpression of miR-451 may reflect a phenotypical resemblance between BSCSs, which are the postulated origin cells of lung adenocarcinoma, and early-stage lung adenocarcinoma.

Several lines of evidence indicate that *MIF* is a candidate target gene of miR-451 and promotes tumor progression [12, 18, 20, 21]. The present results corroborate the evidence by showing that the MIF expression was negatively correlated with the miR-451 expression in NSCLC cases and that a higher MIF expression was correlated with an advanced disease stage. Cell biological experiments of multiple NSCLC cell lines also illustrated the suppression of MIF by the transfection of miR-451-mimic. The increased MIF expression of tumor cells promotes tumor growth through the activation of MAPK/PI3K/Akt pathways by the autocrine or paracrine loop [20]. In fact, the decrease of phosphorylated Akt expression by miR-451-mimic transfection was observed alongside of MIF down-regulation in NSCLC cell lines, while phosphorylated Erk unchanged. The supression of MIF and PI3/Akt pathway would be certainly involved in tumor suppressive role of miR-451. However, the biological role of MIF in NSCLC remains unclear, as a higher MIF expression was not a worse prognostic factor in the analysis. In the loop, the binding of MIF to its receptor, CD74, is indispensable for the activation of MAPK/PI3K/Akt pathways. Therefore, clarifying the biological role of MIF in NSCLC cases will require the evaluation of CD74 status, including the assessment of the recently identified oncogenic fusion gene CD74-NRG1 [32]. By miR-451-mimic transfection, MIF expression was suppressed in NSCLC cell lines. However, we cannot rule out the possibility that the suppression was an indirect effect of miR-451. In our present study, experiments to confirm the direct targeting of miR-451 on *MIF*, such as luciferase reporter assay or an RNA immunoprecipitation (RIP) assay, were not performed, which may limit the validity of the discussion.

In clinical settings of oncology, miRNAs are expected to play roles such as prognostification tools, ancillary markers for a histopathological diagnosis and blood biomarkers [5, 8, 19]. As shown in this study, miR-451 was an independent prognostic marker in NSCLC and is a good candidate for an NSCLC prognostification tool. For histopathological classification, miR-451 was differently expressed between adenocarcinoma and squamous cell carcinoma and may serve as an ancillary marker for the differential diagnosis of adenocarcinoma and squamous cell carcinoma, which is an urgent requirement for the selection of chemotherapy regimens and of molecular-targeted therapy regimens [22]. However, the miR-451 expression varied not only among histopathological types but also among disease stage. Furthermore, the results were based on the analysis of surgically resected samples and were not validated in small biopsy samples. Therefore, further studies are required, such as determining the appropriate threshold for eliminating the influence of disease stage and validating the results in small biopsy samples, before the miR-451 expression can be used as an ancillary marker to differentiate lung adenocarcinoma and small cell carcinoma.

## Conclusions

We analyzed 370 NSCLC cases for their miR-451 expression and found that (1) the miR-451 expression was lower in carcinoma tissues than in paired background lung tissues, (2) a lower expression of miR-451 was an independent predictor of a poor prognosis, and (3) the MIF expression was inversely correlated with the miR-451 expression. It was also shown that MIF expression, phosphorylated Akt expression, cell proliferation, and cell migration were suppressed by miR-451-mimic transfection NSCLC cell lines. This study illustrated the tumor-suppressive role of miR-451 and validated *MIF* as a target of miR-451 in NSCLC from clinicopathological and cell biological points of view.
