# Expression of Concern: The low expression of miR-451 predicts a worse prognosis in non-small cell lung cancer cases

**DOI:** 10.1371/journal.pone.0280774

**Published:** 2023-01-18

**Authors:** The PLOS ONE Editors

The Funding Statement for this article [1] states that the study received funding from the Smoking Research Foundation, which according to [2] has received financial support from the tobacco industry. In light of this issue, *PLOS ONE* has concerns about the article’s [1] compliance with the journal’s policy on Funding from Tobacco Companies [3], which was implemented in 2010.

In response to this issue, the corresponding author stated that, according to the available accounting records, the Smoking Research Foundation funds were used to support a different study and did not contribute to salary or research costs for the study reported in [1]. However, PLOS cannot independently verify this information, and it is unclear whether the accounting records referenced by the author include complete details of the research group’s Smoking Research Foundation fund usage throughout the duration of the study and the article’s peer review.

The *PLOS ONE* Editors issue this Expression of Concern to notify readers of this unresolved issue about the study’s funding.

We regret that this concern was not identified and addressed prior to the article’s [1] publication.
